# Improvements in Bone Disorganization and Pseudo-Fracture Healing in Hypophosphatasia Following Asfotase Alfa Therapy May Be Detectable by the ALIGNOGRAM Before Changes in Bone Radiography or Scintigraphy

**DOI:** 10.1155/crie/5583096

**Published:** 2025-07-13

**Authors:** Roger Zebaze, Simon Zhang, Cat Shore-Lorenti, Cherie Chiang, Frances Milat, Peter Ebeling

**Affiliations:** ^1^Department of Medicine, School of Clinical Sciences, Monash University, Clayton, Victoria, Australia; ^2^Department of Endocrinology, Monash Health, Clayton, Victoria, Australia; ^3^Department of Medicine, Austin Health, University of Melbourne, Heidelberg, Victoria, Australia; ^4^Centre for Endocrinology and Metabolism, Hudson Institute of Medical Research, Clayton, Victoria, Australia

**Keywords:** asfotase alfa, bone disorganization, bone imaging, hypophosphatasia, pseudo-fracture healing

## Abstract

Hypophosphatasia (HPP) is a rare genetic disorder characterized by bone fragility due to defective bone mineralization resulting in impaired alignment of bone components (bone disorganization). Current challenges in the management of HPP include accurately quantifying bone disease severity, monitoring disease progression, and assessing treatment response, particularly in pseudo-fracture healing. Conventional tools such as bone mineral density (BMD) and architecture assessments do not adequately address these issues as they do not assess bone disorganization, limiting their utility in HPP. In this study, we use the ALIGNOGRAM software to reanalyze pseudo-fracture healing in a previously presented case report of an 18-year-old female with benign HPP treated with asfotase alfa. We show that improvements in bone disorganization were detectable as early as 3 months after treatment initiation—before changes on X-ray or bone scintigraphy were detected. As such, assessment of the degree of bone disorganization could serve as an early indicator of treatment efficacy in HPP.

## 1. Introduction

Hypophosphatasia (HPP) is a genetic disease caused by a deficiency of tissue nonspecific alkaline phosphatase (TNAP or TNSALP) activity. Although rare, it is associated with significant morbidity and mortality [1–3]. In adults, HPP presents with heterogenous clinical features and complications. Many patients may remain largely asymptomatic [4], contributing to a delay in diagnosis (median time of 5.7 years) [5]. Upon suspicion, the diagnosis is usually confirmed via clinical, biochemical, and radiological findings [4–6]. Laboratory abnormalities include persistently low serum alkaline phosphatase (ALP) activity, pathogenic ALPL variants and elevated natural substrates such as pyridoxal-5'-phosphate (PLP). To aid with diagnostic accuracy, the International HPP Working Group has developed a standardized diagnostic criteria [6].

While timely diagnosis of HPP remains challenging, an even greater challenge lies in quantifying HPP severity, disease progression, and evaluating treatment response. Biochemical markers used for the diagnosis of HPP are of limited value for monitoring the clinical progression of the disease [7]. Moreover, serum ALP is nonspecific and may also be low in subjects with hypoparathyroidism or those on antiresorptive agents [8]. Current tools for assessing HPP severity and response to therapy, such as the 6-Minute Walk Test (6MWT), sit-to-stand (STS) test, Timed Up-and-Go (TUG) test, short physical performance battery (SPPB), handheld dynamometry (HHD), and the Lower Extremity Functional Scale (LEFS), are predominantly measures of physical function and health-related quality of life (HRQoL) [6, 7]. Whilst useful, these tests may be affected by factors unrelated to HPP, such as cardiovascular and musculoskeletal comorbidities, nutritional status, and cognitive function [9].

Given HPP is fundamentally a disorder of mineralization and bone abnormalities are one of its most serious manifestations, the bone represents an ideal target for a diagnostic modality [6, 10]. Conventional bone parameters such as bone mineral density (BMD) can be normal or even elevated in patients with HPP and thus are of limited value [11–15]. Meanwhile, measurements of bone architecture using novel approaches such as quantitative computed tomography (QCT), and more recently, the advanced high-resolution peripheral QCT (HRpQCT) have yet to demonstrate clear evidence of structural abnormalities or reduced bone strength (estimated using finite element analysis) in HPP patients [14]. Thus, there is a need to identify another distinct bone parameter in HPP.

We recently reported that for bone to be structurally sound, its components must be organized and correctly aligned with the “the right bone component at the right position.” This is a critical yet often overlooked feature of bone [15]. In HPP, we propose that bone disease results from loss-of-function mutations in ALPL which disrupt mineralization, leading to an impaired organization (arrangement, alignment) of bone components [15]. Consequently, quantifying the extent of bone disorganization may offer a potentially sensitive and specific marker of disease severity, progression, and therapeutic improvement in HPP. We recently developed and validated a method of quantifying the extent of disorganization of bone components using the ALIGNOGRAM_1.0_ software. This tool uses standard, readily available X-rays, making it widely accessible [16].

Asfotase alfa (Strensiq, Alexion Pharmaceuticals Inc., Boston, MA, USA) is currently the only approved treatment for HPP. We propose that this drug improves bone mineralization which leads to an improvement in bone disorganization (misassembly). Hence, measuring bone disorganization should represent a robust parameter for assessing the severity of HPP and monitoring improvements during treatment with asfotase alfa [3]. We quantified this novel bone property using the ALIGNOGRAM_1.0_ in an 18-year-old female with benign prenatal HPP from a case report originally published by Kato et al. [3]. Correlations and comparisons between disorganization measurements and other available published data (X-rays, scintigraphy, physical function, and laboratory markers) were made.

## 2. Case Presentation

This case is based on the reanalysis of the images obtained from Kato et al. [3]. Although the manuscript was published under creativecommons.org/licenses/by/4.0/, permission to reuse the images and the article content was obtained from the publisher. Left tibial X-ray and bone scintigraphy were performed at baseline, and at regular intervals of 3, 6, 12, 24, and 48 months after initiation of asfotase alfa in an 18-year-old Japanese female who was diagnosed with benign prenatal HPP. At age 17, she sustained a left tibial pseudo-fracture. The details about the clinical presentation, clinical manifestations, phenotype, genotype, biochemistry, and functional assessment are described in detail in Kato et al. [3].

**Figure 1:**
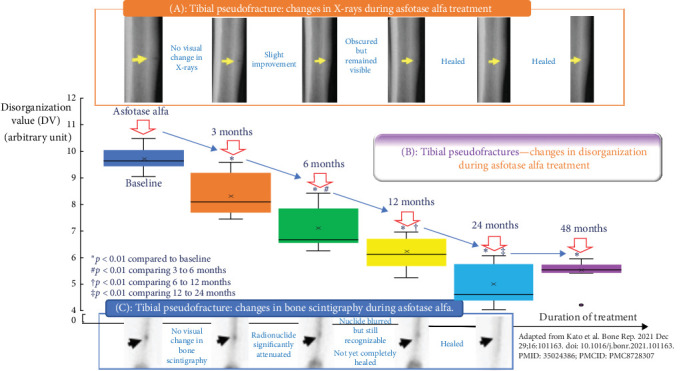
(A, upper panel): Radiographic (X-rays) changes of a pseudo-fracture in an 18-year-old Japanese female with benign prenatal HPP following asfotase alfa treatment. Improvements are only noticeable after 6 months of treatment. Healing is observed at 2 years. (B, middle panel): Box and Whisker plots showing disorganization values before the initiation of asfotase alfa (dark blue), at 3 months after starting treatment (orange), then at 6 months (green), 12 months (yellow), after 1 year (light blue), and after 2 years (purple). Note that there is improvement in disorganization as early as 3 months after treatment initiation. This is visible at the time when X-rays and bone scintigraphy do not yet show any changes. Furthermore, there is continuous and significant improvement in the magnitude of bone disorganization noticeable at each consecutive timeline until complete healing at 24 months. (C, lower): Bone scintigraphy changes of a pseudo-fracture in the adult patient with benign HPP following asfotase alfa treatment. Improvements are only noticeable after 6 months of treatment. Healing is observed at 2 years.

**Figure 2:**
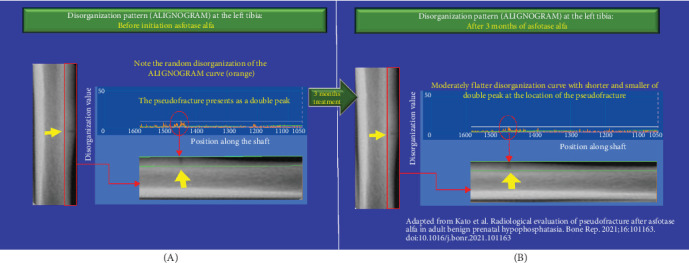
(A, B) Improvement in the disorganization pattern (ALIGNOGRAM curve) at the left tibia following 3 months of asfotase alfa treatment. Left panels are portions of left tibia showing the pseudo-fracture. Right lower panels are the same portions of the left tibia with the corresponding ALIGNOGRAMs overlaying on top (right upper panels). The location analyzed is shown as a green rectangle. The ALIGNOGRAM shows the quantified disorganization (misarrangement, misalignment) values on the *Y*-axis and the position along the bone (tibia) in pixels on the *X*-axis. The green line shows the expected disorganization values at each position. The white line is the threshold value above which any disorganization value is considered an outlier. (A) The orange curve displays the disorganization values (DVs) at each position along the shaft before initiation of asfotase alfa. The double peak corresponding to the pseudo-fracture is clearly visible. In addition, small peaks with excessively high disorganized values particularly near the location corresponding to the pseudo-fracture are also visible. (B) After 3 months of treatment, the disorganization pattern (orange curve) is flatter and more regular. Noticeably, the area with the double peak corresponding to the pseudo-fracture is smaller and shorter. Small, short peaks are due to physiological background disorganization.

We assessed bone disorganization using the ALIGNOGRAM at each interval in which X-ray and bone scintigraphy were performed by Kato et al. (Figure 1). The ALIGNOGRAM curve displays the position along the bone on the X-axis and the magnitude of disorganization, represented as the disorganization value (DV; arbitrary unit), on the Y-axis. The lateral cortex is subdivided into nine consecutive rectangular regions of interest (ROI) to allow a detailed assessment of bone disorganization characteristics. At each location along the bone, the pattern of disorganization is quantified and displayed as an orange curve (Figure 2).

At baseline, prior to the commencement of asfotase alfa, the ALIGNOGRAM showed a distinct double peak of high DVs at the location corresponding to the pseudo-fracture (Figure 2). In addition, there are small sharp peaks with excessively disorganized values in locations adjacent to the pseudo-fracture. As published, X-ray imaging and bone scintigraphy confirmed the pseudo-fracture [3].

After 3 months of asfotase alfa treatment, analysis using the ALIGNOGRAM demonstrated a significant reduction in bone disorganization, with a 15.97% decrease in the median DV from 9.64 (IQR 9.56–9.91) to 8.10 (IQR 7.84–8.91) (*p*  < 0.001) (B). Figure 2 shows that the disorganization curve was more regular and flatter, while the double peak with high DVs at the location corresponding to the pseudo-fracture was also shorter and smaller. As published by Kato et al. [3], no visible changes in pseudo-fracture healing were noted on X-ray (Figure 1A) or bone scintigraphy (Figure 1C).

**Figure 3:**
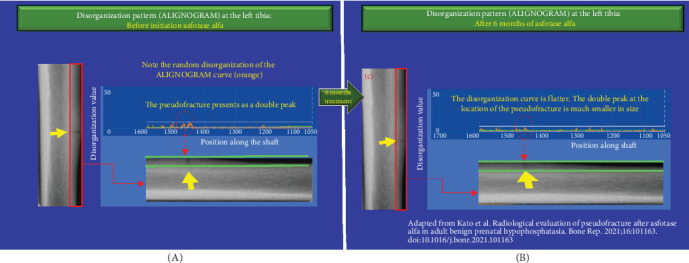
(A, B) Improvement in the disorganization pattern (ALIGNOGRAM curve) at the left tibia following 6 months of asfotase alfa treatment. (A) The orange curve displays the pattern of disorganization values (DVs) at each position along the shaft before the initiation of asfotase alfa. The double peak corresponding to the pseudo-fracture is visible as described in Figure 2A. (B) Compared to baseline, after 6 months of treatment, the disorganization pattern (orange curve) is much flatter and more regular. Noticeably, the area with the double peak corresponding to the pseudo-fracture is much smaller and shorter. Small, short peaks are due to physiological background disorganization.

At 6 months postinitiation of asfotase alfa therapy, there was further improvement in the extent of disorganization on our analysis using the ALIGNOGRAM, with a 17.53% decrease in the median DV from 8.10 (IQR 7.84–8.91) to 6.68 (IQR 6.56–7.57) (*p*  < 0.01) between 3 and 6 months, respectively. As shown in Figure 3, there was a further flattening of the disorganization curve with a smaller and shorter double peak corresponding to the healing pseudo-fracture (Figure 1C) [3]. Quantitative assessment of bone disorganization has already demonstrated a marked improvement in median DV from baseline (30.7%; *p*  < 0.0001) (Figure 1). It was at this time that pseudo-fracture healing has first became noticeable on X-ray and bone scintigraphy [3]. In light of the noted improvements, Kato et al. [3] reduced the dose of asfotase alfa to 80 mg twice weekly (4 mg/kg/week).

**Figure 4:**
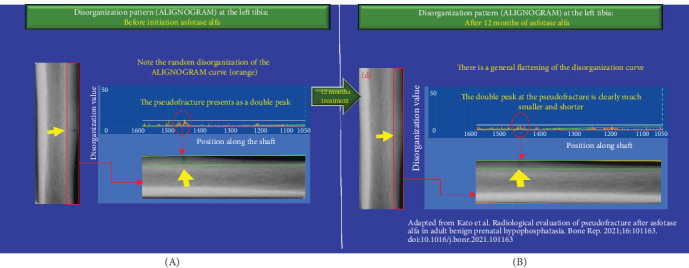
(A, B) Improvement in the disorganization pattern (ALIGNOGRAM curve) at the left tibia following 12 months of Asfotase treatment. (A) The orange curve displays the pattern of disorganization values (DVs) at each position along the shaft before the initiation of asfotase alfa. The double peak corresponding to the pseudo-fracture is visible as described in Figure 2A. (B) Compared to baseline, after 12 months of treatment, there is a significantly smaller and shorter double peak corresponding to the pseudo-fracture. The overall disorganization curve (orange) is also flatter and regular. Small, short peaks are due to physiological background disorganization.

At 12 months postinitiation of asfotase alfa therapy, we report continuous quantitative improvement in the extent of disorganization on the ALIGNOGRAM between 6 and 12 months, with an 8.38% improvement in the median DV from 6.68 (IQR 6.56–7.57) versus 6.12 (IQR 6.07–6.69) (*p*  < 0.05) (Figure 1B, middle panel). There was more flattening of the disorganization curve and further reduction in the size of the double peak corresponding to the pseudo-fracture (Figure 4). At this point, when pseudo-fracture healing can clearly be visualized on standard imaging, quantitative assessment of bone disorganization has already revealed a marked 36.5% (*p*  < 0.0001) decrease in disorganization from baseline (Figure 1). Kato et al. further reported that it is at this stage that changes in physical function tests (6MWT and TUG) had become noticeable.

**Figure 5:**
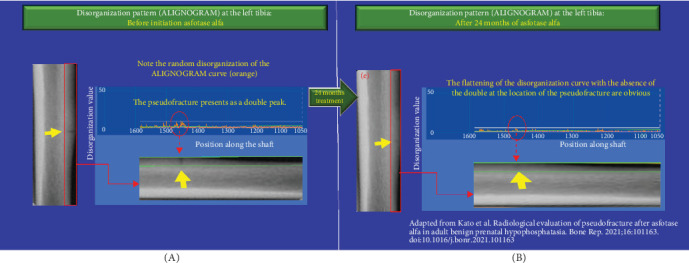
(A, B) Improvement in the disorganization pattern (ALIGNOGRAM curve) at the left tibia following 24 months of Asfotase treatment. (A) The orange curve displays the pattern of disorganization values (DVs) at each position along the shaft before the initiation of asfotase alfa. The double peak corresponding to the pseudo-fracture is visible as described in Figure 2A. (B) Compared to baseline, after 24 months of treatment, there is marked improvement in the disorganization with flattening in the disorganization curve clearly visible. No disorganization value reaches the outlier threshold (white line). The double peak with high disorganization values at the location corresponding to the pseudo-fracture had nearly disappeared. Small, short peaks are due to physiological background disorganization.

At the 24-month interval, we report an additional marked improvement in the disorganization with a continuous flattening in the disorganization curve. The double peak with high DVs at the location corresponding to the pseudo-fracture has nearly disappeared (Figure 5). Quantitatively, the additional improvement in the extent of bone material disorganization was remarkable (24.6%), with the median DV further decreasing from 6.12 (IQR 6.07–6.69) vs 4.61 (IQR 4.53–5.5) (*p*  < 0.0001) (Figure 1B, middle panel). In sum, during the first 2 years of treatment with asfotase alfa, there was a continuous and sustained decrease in the extent of disorganization with bone organization improving by a total of 52.17% (*p*  < 0.0001) (Figure 1). X-ray and bone scintigraphy (Figure 1A) confirm that the pseudo-fracture had completely disappeared as published [3].

**Figure 6:**
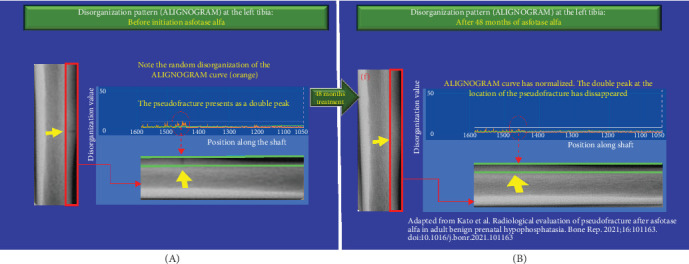
(A, B) Improvement in the disorganization pattern (ALIGNOGRAM curve) at the left tibia following 48 months of Asfotase treatment. (A) The orange curve displays the pattern of disorganization values (DVs) at each position along the shaft before the initiation of asfotase alfa. The double peak corresponding to the pseudo-fracture is visible as described in Figure 2A. (B) Compared to baseline, after 48 months of treatment, there is improvement in bone quality (organization) once again, as shown by a nearly flat orange curve compared to baseline. Only background physiological disorganization remained. The double peak corresponding to the pseudo-fracture had disappeared. Small, short peaks are due to physiological background disorganization.

**Figure 7:**
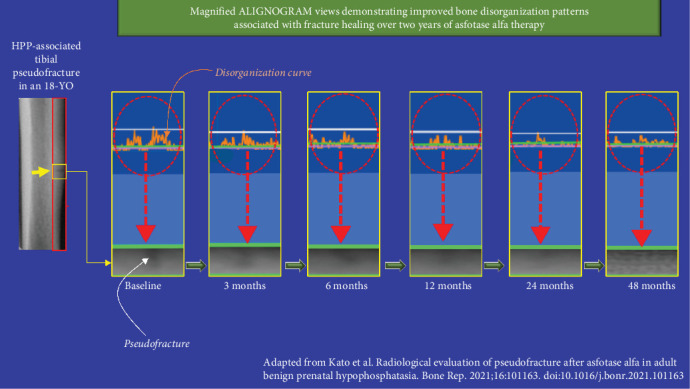
A segment of the tibia from an 18-year-old Japanese female with benign prenatal hypophosphatasia (HPP) and a pseudofracture is shown on the far left. The yellow-bordered rectangles highlight the disorganization pattern (top, orange curves) and the corresponding pseudofracture site (bottom). Improvement in bone structure is evidenced by the flattening of the disorganization curves as the pseudofracture heals.

At 48 months after commencing asfotase alfa, the improvement in bone quality (organization) was maintained—as shown by a nearly flat curve with only background physiological disorganization remaining. The double peak corresponding to the pseudo fracture had disappeared (Figure 6). No further improvement in the extent of disorganization was noted between 2 and 4 years from baseline (Figure 1B, middle panel). Complete union and healing of the pseudo-fracture on X-ray (Figure 1A; Kato et al., Figure 2) and bone scintigraphy (Figure 1C; Kato et al., Figure 2) are confirmed in the original publication [3].  Figure 7 provides magnified views of key areas to better illustrate the observed improvements across the 48 months of asfotase alfa treatment.

**Figure 8:**
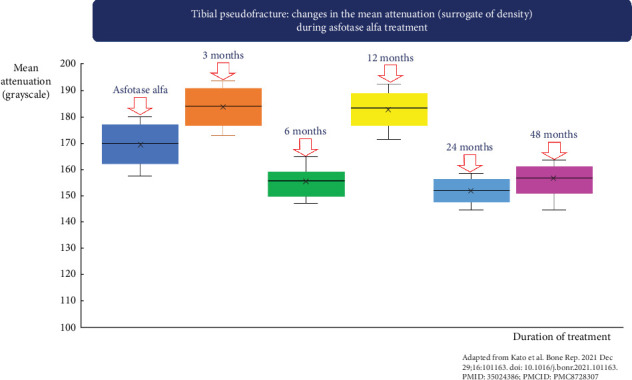
Box and Whisker plots showing mean attenuation values (surrogate of density) before the initiation of Asfostase Alfa (dark blue), at 3 months after starting treatment (orange), at 6 months (green), at 12 months (yellow), at 1 year (light blue), and at 2 years (purple). Note that there was no relationship between the duration of Asfotase treatment and the mean attenuation value, a surrogate of bone density in the region of the pseudo-fracture where disorganization measurements were performed.

The mean attenuation values, which serve as a surrogate of bone density, were also assessed using the ALIGNOGRAM for the duration of asfotase alfa therapy. We found no changes in the mean attenuation values in the region where disorganization was measured (Figure 8).

## 3. Discussion

This is the first study to provide a detailed description of changes in bone organization (arrangement, alignment) during asfotase alfa therapy in an adult patient with benign prenatal HPP. Evaluation of the patient's pseudo-fracture was performed using the ALIGNOGRAM at baseline and at 3, 6, 12, 24, and 48 months after initiation of asfotase alfa. This was in addition to the X-ray and bone scintigraphy assessments reported by Kato et al. [3].

Quantification of bone disease severity, its progression, as well as response to therapy remains a significant challenge in the management of patients with adult HPP. Typical methods using BMD and bone microarchitecture are of limited value, given bone health is not solely determined by its mass, density or structure [11–15]. Indeed, bone which has normal or high BMD, and no structural decay does not necessarily imply that the bone is healthy. The structural elements of bone must also be properly assembled and aligned to ensure optimal cohesion and structural stability when subjected to a load [16]. This may be exemplified by using the analogy of a Jenga tower, where although the total number of blocks may be sufficient (normal density) and each individual block may be structurally sound (normal architecture), the entire structure may ultimately become unstable and collapse if a block is placed in the wrong location or removed from where it should remain. Bone disorganization describes the misarrangement of bone components in space and leads to ineffective load conduction of forces through bone tissue and consequently, bone abnormalities such as fragility fractures [16]. Bone diseases which are characterized by bone disorganization include HPP, osteogenesis imperfecta, osteopetrosis, chronic kidney disease metabolic bone disorder, neurofibromatosis, diabetes mellitus, and antiresorptive therapy-associated AFFs [16–18]. In these diseases, bone markers such as density and structure are typically normal or not markedly impaired [12–14]. In this context, the ALIGNOGRAM is a novel tool which can quantify disorganization by measuring the misalignment of bone components in such diseases, as well as assess the dynamic process of fracture healing such as that in our patients using serial standard X-rays [16].

In this study, the baseline DV of 9.64 in this patient's pseudofracture falls within the range previously reported in patients with atypical femoral fractures (AFFs; 5–15 DV). After 24 months of treatment, the DV in this patient decreased to ~4, a level consistent with that observed in control subjects without AFFs [16]. An improvement in bone disorganization and healing of the pseudo-fracture was detectable at 3 months, before both bone scintigraphy (an advanced and more expensive technology) and visual assessment of X-rays. This early therapeutic response was supported by the clinical improvement in pain which occurred within 2 months and urinary PEA level which decreased as early as 1 month after therapy [3]. While pain improvement is a common clinical indicator of fracture healing, its nonspecific nature necessitates confirmatory imaging at the fracture site. Objective assessment using standard X-rays or advanced modalities (MRI, CT, or scintigraphy) remains essential for anatomical validation. Our findings suggest that novel tools capable of quantifying bone misalignment—such as the ALIGNOGRAM—may detect therapeutic response earlier than conventional radiography and, in some cases, even bone scintigraphy. This advantage stems from their ability to detect microstructural disruptions and disorganization, which are fundamental features of fractures often invisible to traditional imaging. It should be noted that the patient demonstrated delayed healing, marked by persistent severe left tibial pain for over a year and a visible baseline fracture line prior to asfotase alfa therapy. The concurrent improvements in pain, biochemical markers, radiographic healing, and ALIGNOGRAM-quantified reduction in bone disorganization following treatment initiation collectively indicate treatment-induced healing, rather than spontaneous resolution [3].

The ability of the disorganization measurement technology to detect early changes in patients with HPP is supported by mechanistic and technological considerations. Mechanistically, we hypothesize that defective bone mineralization in HPP leads to an impaired bone organization. Consequently, effective therapy which corrects the underlying mineralization defect should also improve bone organization [15]. From a technological perspective, the ALIGNOGRAM tool performs a pixel-by-pixel analysis of bone disorganization, comparing each pixel to the adjacent pixels. The degree of dissimilarity or deviation of a pixel from its neighbors, reflecting local disorganization, is quantified to determine the extent of bone disorganization [16]. As previously described, this pixel-by-pixel analysis enables the detection of disorganization changes as small as a single pixel—an abnormality which may not be visible to the naked eye [16]. This capability of the ALIGNOGRAM may explain the early detection of the therapeutic response in this patient, where other methods have failed to detect any improvements.

The early radiological detection of pseudo-fracture healing has been previously described. Kildaras et al. reported callus formation at the osteotomy site within 2 months of initiating asfotase alfa, with a definite increase in bridging callus formation observed after 6 months [17]. Similarly, Genest et al. reported initial signs of consolidation at 3 months and complete osseous bridging at the fracture site after 6 months of follow-up [19]. This study builds upon these findings by providing a quantitative measurement of the early therapeutic effects of asfotase alfa treatment. If validated in future studies, tools like the ALIGNOGRAM could enable clinicians to assess fracture healing within 3 months of intervention, eliminating the need to wait for visible repair on standard X-rays or bone scintigraphy. This paradigm shift could accelerate clinical decision-making and support more personalized therapeutic strategies for patients with delayed healing.

The improvement in bone disorganization was sustained throughout the first 2 years of treatment. Whilst differences in disorganization levels were clearly visible and significant at each time point in treatment, changes observed on X-ray or bone scintigraphy were more difficult to appreciate. After 1 year of treatment, when bone scintigraphy and X-rays began to show signs of pseudo-fracture healing, a substantial improvement in bone disorganization (~30%) was already detectable (Figure 1). After 2 years, as the pseudo-fracture had fully healed on X-ray and bone scintigraphy, only background physiological bone disorganization remained, confirming the restoration of good bone organization and successful pseudo-fracture healing with therapy. It should be noted that bone is an inherently heterogeneous material, and this physiological variability contributes to a baseline level of disorganization.

The assessment of bone disorganization is likely a useful complement in the investigation and management of patients with HPP. Notably, commonly used clinical endpoints to assess response to the therapy such as the 6MWT and TUG did not show significant improvements even after 12 months of treatment [3]. In contrast, the improvement in bone disorganization was substantial and evident at this time. It is possible that when baseline physical function test results are minimally or moderately impaired, the extent of their improvement with therapy may also be modest, as observed in this patient [3]. Only the STS and weighted arm lift test improved after 12 months [3]. Whether these measures could have demonstrated earlier improvements is unclear, as these data were not available. It should be noted that Rolvien et al. [19] reported that muscle function measured using the chair rising test and grip strength test improved after 8 and 16 months of treatment with asfotase alfa. Genest et al. [20] also reported significant improvements in chair-rise time and SF-36v2 physical component summary (PCS) after 3 months, TUG test time after 6 months, and 6 MWT after 12 months of treatment with asfotase alfa.

BMD measurement is not recommended in patients with HPP, as it provides minimal clinical utility and does not assess osteomalacia [21]. Accordingly, BMD is excluded from current diagnostic guidelines for HPP [5]. In this study, no relationship between attenuation value (a surrogate of density) and treatment was found. Interestingly, Rolvien et al. [19] observed a moderate improvement in BMD after 12 months of therapy. However, it should be noted that the relationship between BMD and bone fragility is complex, and a higher BMD does not necessarily equate to lower fracture risk, particularly in patients with HPP [11, 12]. Indeed, patients with HPP often have normal BMD unless they have concomitant osteoporosis secondary to other causes [12, 21]. Beyond BMD, Rolvien et al. [19] reported that bone microarchitecture assessed using HRpQCT remained relatively constant after 12 months of asfotase alfa. Notably, they did not find any microstructural changes at the distal radius [19].

This study has several limitations. Firstly, only a single HPP patient undergoing treatment was analyzed. However, as an initial pilot study employing a novel approach, this serves as a foundational investigation. Future prospective studies are needed to support these findings. Secondly, the clinical course of the patient in this case may not depict the typical pseudo-fracture healing process seen in other patients with HPP treated with asfotase alfa. This may be due to variability such as fracture location, type of HPP, age, sex, and physical activity level. Nevertheless, although the patient presented in this report had a relatively benign form of HPP, early changes were still detectable after treatment initiation. Thirdly, this study relied on open-source material. While this may be seen as a limitation, the use of open-source material avoided the unnecessary need for additional experiments which would have imposed considerable costs, time, logistical challenges, and patient inconvenience. Given that this is a rare disease, conducting similar studies including 4 years of follow-up data would have been challenging.

In summary, this study demonstrated for the first time that disorganization, a novel imaging parameter, can detect skeletal defects in HPP as well as the therapeutic effectiveness of asfotase alfa therapy prior to the appearance of visible changes in bone scintigraphy or X-ray. Furthermore, these improvements were sustained and continuous throughout the first 2 years of therapy. These findings suggest that bone disorganization may serve as a valuable tool to complement other assessments such as changes in physical function and radiological findings. As disorganization can be quantified from simple, standard widely available X-rays, it has the potential to provide an accessible and transformative approach to monitoring HPP treatment. Studies with larger cohorts of HPP patients treated with asfotase alfa treatment are required to confirm our findings.

## Data Availability

The data that support the findings of this study are available from the corresponding author upon reasonable request.

## References

[1] Tournis S., Yavropoulou M. P., Polyzos S. A., Doulgeraki A. (2021). Hypophosphatasia. *Journal of Clinical Medicine*.

[2] Mornet E. (2017). Genetics of Hypophosphatasia. *Archives de Pédiatrie*.

[3] Kato H., Hidaka N., Koga M. (2022). Radiological Evaluation of Pseudofracture After the Administration of Asfotase Alfa in an Adult With Benign Prenatal Hypophosphatasia: A Case Report. *Bone Reports*.

[4] Michałus I., Gawlik A., Wieczorek-Szukała K., Lewiński A. (2022). The Clinical Picture of Patients Suffering From Hypophosphatasia—A Rare Metabolic Disease of Many Faces. *Diagnostics*.

[5] Khan A. A., Brandi M. L., Rush E. T. (2024). Hypophosphatasia Diagnosis: Current State of the Art and Proposed Diagnostic Criteria for Children and Adults. *Osteoporosis International*.

[6] Rush E., Brandi M. L., Khan A. (2024). Proposed Diagnostic Criteria for the Diagnosis of Hypophosphatasia in Children and Adolescents: Results From the HPP International Working Group. *Osteoporosis International*.

[7] Kishnani P. S., Rush E. T., Arundel P. (2017). Monitoring Guidance for Patients With Hypophosphatasia Treated With Asfotase Alfa. *Molecular Genetics and Metabolism*.

[8] Bertoldo F., Tripepi G., Zaninotto M. (2025). Possible Role of Bone Turnover Markers in the Diagnosis of Adult Hypophosphatasia. *Journal of Bone and Mineral Research*.

[9] Heresi G. A., Dweik R. A. (2011). Strengths and Limitations of the 6-Minute-Walk Test: A Model Biomarker Study in Idiopathic Pulmonary Fibrosis. *American Journal of Respiratory and Critical Care Medicine*.

[10] Barvencik F., Beil F. T., Gebauer M. (2011). Skeletal Mineralization Defects in Adult Hypophosphatasia—A Clinical and Histological Analysis. *Osteoporosis International*.

[11] Siris E. S., Miller P. D., Barrett-Connor E. (2001). Identification and Fracture Outcomes of Undiagnosed Low Bone Mineral Density in Postmenopausal Women: Results From the National Osteoporosis Risk Assessment. *JAMA*.

[12] Genest F., Claußen L., Rak D., Seefried L. (2021). Bone Mineral Density and Fracture Risk in Adult Patients With Hypophosphatasia. *Osteoporosis International*.

[13] Carazo S. G., Hernández C. L., Criado A. B., Acín P. A. (2020). Evaluation of Bone Mineral Density and 3D-Shaper Parameters in Congenital Hypophosphatasia of the Adult. *Journal of Osteoporosis & Mineral Metabolism*.

[14] Sidhu K., Ali B., Burt L. A., Boyd S. K., Khan A. (2020). Spectrum of Microarchitectural Bone Disease in Inborn Errors of Metabolism: A Cross-Sectional, Observational Study. *Orphanet Journal of Rare Diseases*.

[15] Zebaze R., Ebeling P. R. (2023). Disorganization and Musculoskeletal Diseases: Novel Insights Into the Enigma of Unexplained Bone Abnormalities and Fragility Fractures. *Current Osteoporosis Reports*.

[16] Zebaze R., Shore-Lorenti C., Nguyen H. H., Chiang C., Milat F., Ebeling P. R. (2023). A Quantification Method for Disorganized Bone Components: Application to the Femoral Shaft. *Journal of Bone and Mineral Research Plus*.

[17] Klidaras P., Severt J., Aggers D., Payne J., Miller P. D., Ing S. W. (2018). Fracture Healing in Two Adult Patients With Hypophosphatasia After Asfotase Alfa Therapy. *Journal of Bone and Mineral Research Plus*.

[18] Zhang S. C., Makebeh T., Mesinovic J. (2024). Epidemiology of Fractures in Adults of African Ancestry With Diabetes Mellitus: A Systematic Review and Meta-Analysis. *Bone*.

[19] Rolvien T., Schmidt T., Schmidt F. N. (2019). Recovery of Bone Mineralization and Quality During Asfotase Alfa Treatment in an Adult Patient With Infantile-Onset Hypophosphatasia. *Bone*.

[20] Genest F., Rak D., Petryk A., Seefried L. (2020). Physical Function and Health Related Quality of Life in Adults Treated With Asfotase Alfa for Pediatric Onset Hypophosphatasia. *Journal of Bone and Mineral Research Plus*.

[21] Simmons J. H., Rush E. T., Petryk A., Zhou S., Martos-Moreno G.Á. (2020). Dual X-ray Absorptiometry Has Limited Utility in Detecting Bone Pathology in Children With Hypophosphatasia: A Pooled Post Hoc Analysis of Asfotase Alfa Clinical Trial Data. *Bone*.

